# Catheter-Directed Thrombolysis vs. Anticoagulation in Deep Vein Thrombosis: A Comparative Study

**DOI:** 10.3390/jcm14103298

**Published:** 2025-05-09

**Authors:** Mehmet Cahit Saricaoglu, Ali Ihsan Hasde, Ali Fuat Karacuha, Ahmet Kayan, Onur Buyukcakır, Fatma Akca, Evren Ozcinar, Cagdas Baran, Mustafa Bahadir Inan, Mustafa Sirlak, Levent Yazicioglu, Ahmet Ruchan Akar, Sadik Eryilmaz

**Affiliations:** 1 Department of Cardiovascular Surgery, Heart Center, Cebeci Hospitals, Ankara University School of Medicine, 06340 Ankara, Turkey; cahitsarica@gmail.com (M.C.S.); dr.ahmet.kayan@gmail.com (A.K.); buyukcakironur@gmail.com (O.B.); evrenozcinar@gmail.com (E.O.); cagdasbaran@gmail.com (C.B.); mbahadirinan@gmail.com (M.B.I.); mustafasirlak@gmail.com (M.S.); leventyazicioglu@gmail.com (L.Y.); akarruchan@gmail.com (A.R.A.); sadikeryilmaz@gmail.com (S.E.); 2 Department of Cardiovascular Surgery, Trabzon Kanuni Education and Research Hospital, 61250 Trabzon, Turkey; alifuatkaracuha@hotmail.com; 3 Department of Cardiovascular Surgery, Kirikkale High Specialization Hospital, 71300 Kirikkale, Turkey; akcaafatma@gmail.com

**Keywords:** catheter-directed thrombolysis, deep vein thromboembolism, anticoagulation

## Abstract

**Background:** Deep vein thrombosis (DVT) is an important component of venous thromboembolism and can lead to pulmonary embolism with high morbidity and mortality. Anticoagulant therapy alone (AC) and catheter-directed thrombolysis (CDT) are commonly used strategies for the management of DVT. Although CDT has been reported to be effective in reducing the risk of post-thrombotic syndrome (PTS), it remains unclear in which patient groups it should be preferred due to the risk of bleeding. **Methods:** This retrospective study included 175 patients diagnosed with DVT between 2015 and 2024 (98 AC, 77 CDT). Patients with a diagnosis of proximal DVT, aged ≥18 years, and with at least 30 days of follow-up data were included. The primary endpoint was 30-day mortality and secondary endpoints were the length of hospitalization, pulmonary embolism, and bleeding complications. **Results:** The CDT group was superior to AC in thrombus clearance rates, especially in iliac vein thrombosis (97.7% vs. 78%, *p* = 0.003). Clinical symptoms improved faster in the CDT group, but total hospitalization was longer. There were no significant differences in bleeding complications and mortality rates between the two groups. **Conclusions:** The optimal approach to DVT treatment should be based on the patient’s individual risk factors. Although CDT provides a higher thrombus clearance rate, especially in iliac vein thrombosis, it may not be suitable for all patients. Future large-scale studies will contribute to a better understanding of the long-term outcomes of interventional therapies.

## 1. Introduction

Deep vein thrombosis (DVT) represents a major clinical manifestation of venous thromboembolism (VTE) and serves as a significant precursor to pulmonary embolism (PE), contributing substantially to both morbidity and mortality [1]. VTE is the third most common cause of death among cardiovascular diseases worldwide and constitutes a significant risk factor, especially for hospitalized patients [2]. The emergence of the COVID-19 pandemic has significantly amplified the clinical burden of VTE, thereby underscoring the urgent need for more effective and evidence-based therapeutic strategies to optimize patient management [1].

Studies on the prevalence and incidence of DVT reveal that the global health impact of this disease is increasing, and risk factors need to be better understood [3]. Current guidelines recommend individualized treatment approaches and emphasize that optimal treatment should be selected based on the patients’ thrombotic burden, symptom duration, and general health status [2]. Recent studies on the efficacy of antithrombotic therapies suggest that combined treatment strategies may be considered to reduce complication rates [4].

The main approaches to the treatment of DVT include anticoagulant therapy (AC) and catheter-directed thrombolysis (CDT). AC therapy prevents the clot from growing, allowing time for the body’s natural fibrinolytic system to break it down, while CDT aims to directly dissolve the thrombus, which may provide faster clinical improvement [2]. However, uncertainties persist concerning the long-term efficacy and safety of both methods. CDT has been reported to preserve the venous function, reduce the risk of post-thrombotic syndrome (PTS) (the “open-vein hypothesis”), and improve quality of life, especially in patients with proximal DVT [5,6]. However, it is still controversial in which patient groups CDT should be preferred because it is a more invasive method and involves an inherent risk of hemorrhage [7].

Management of DVT has come to encompass a broad variety of therapeutic strategies, which include pharmacological anticoagulation and interventions. AC therapy is still the foundation of initial management, with the objective of thwarting thrombus extension and minimizing the hazard of pulmonary embolism by allowing the body’s natural fibrinolytic mechanism to dissolve the clot [4]. Yet, interventional strategies like CDT, mechanical thrombectomy, and pharmaco-mechanical thrombolysis have become more prominent in the last several years, especially in patients presenting with extensive or iliofemoral thrombosis [2]. They enable immediate thrombus removal and can possibly minimize the occurrence of PTS in carefully selected patients [5]. Their use should be individualized, taking into account thrombus burden, duration of symptoms, risk of bleeding, and institutional experience. Catheter-directed thrombolysis is a minimally invasive endovascular procedure whereby a multi-sidehole catheter is placed within the occluded vein under the guidance of ultrasound or fluoroscopy. A thrombolytic agent, usually alteplase, is subsequently infused directly into the thrombus to induce localized breakdown with minimal systemic exposure. CDT may be employed alone or in conjunction with mechanical thrombectomy in certain situations to augment the effectiveness of thrombus removal. Typically, this procedure is carried out as a one-time treatment within a 24 h period, with emphasis on the acute lysis of thrombus, thereby obviating the need for serial or ongoing treatment sessions.

The 2019 European Society of Cardiology (ESC) guidelines also state that CDT may be considered in patients with significant thrombosis who are at high risk for developing PE or PTS, particularly if there is low risk for bleeding and availability of resources [8]. These recommendations highlight the importance of individualized treatment decisions in the management of DVT.

In this study, we compared the clinical outcomes of CDT and AC-only groups in patients diagnosed with DVT. The efficacy and safety of treatment approaches were analyzed. In addition, the development of PTS was descriptively evaluated during follow-up based on clinical findings, although it was not defined as a primary or secondary endpoint due to the retrospective design of the study. Thus, we aimed to provide evidence-based data that may contribute to reducing the global health burden of VTE.

## 2. Material and Methods

### 2.1. Study Design

This was a single-center, retrospective study of patients with DVT who received treatment at Ankara University Hospital from 2015 to 2024. Approval for the study was obtained from the Human Research Ethics Committee of the Ankara University School of Medicine (date: 26 March 2025; no. 2025/269). A total of 175 patients were included in the study, 98 of whom were treated with ACA and 77 with CDT.

Patients who were 18 years of age or older, diagnosed with proximal DVT of the lower extremities, and who had at least 30 days of follow-up data were included in the study. Patients with a stable general condition, low bleeding risk, and a life expectancy of at least one year were included. Both provoked and unprovoked DVT cases were included in the study. DVT etiology was not used as a stratification variable in the primary analysis. Patients who had received thrombolytic therapy for pulmonary embolism, patients with active bleeding or severe bleeding risk, patients with a history of previous lower extremity venous surgery, and patients in pregnancy or the postpartum period were excluded.

### 2.2. Outcomes

The primary endpoint of the study was postoperative 30-day mortality. Secondary endpoints were defined as intensive care unit length of stay, total hospital stay, pulmonary embolism, and major and minor bleeding. While PTS was not a pre-specified primary or secondary outcome, it was evaluated descriptively during follow-up by considering clinical presentation and symptoms. Because of the study’s retrospective nature, a validated scoring system, such as the Villalta scale, was not used uniformly. The Villalta scale is a widely used accepted measure for diagnosing PTS that incorporates patient-reported symptoms and clinical findings to provide a severity score. In lieu, evaluation was centered on limb pain, swelling, skin discoloration, and venous stasis in regular clinical assessments [9]. Major bleeding was classified based on ISTH criteria as bleeding that was fatal, symptomatic intracranial hemorrhage, or bleeding that resulted in hemodynamic instability or necessitated surgical intervention or transfusion of ≥2 units of blood [10]. Clinically relevant non-major bleeding was defined as any overt bleeding that did not meet major criteria but still required medical attention, according to Kaatz et al. [11].

### 2.3. Treatment Protocol

Patients receiving only anticoagulant therapy received intravenous unfractionated heparin infusion. Heparin infusion was administered with dose adjustment so that the activated partial thromboplastin time (aPTT) value was 1.5–2.5 times. Since an anti-Xa assay was not available in our center, aPTT-guided dose adjustment was utilized. In cases of prolonged baseline aPTT (i.e., with lupus anticoagulant present), clinical parameters and coagulation trends were considered to guide therapy. Following initial anticoagulation with intravenous unfractionated heparin, all patients were given full-dose oral anticoagulation therapy, and treatment duration was planned for at least 3 months. The duration of treatment was determined according to the individual thromboembolic risk factors of the patients.

**Figure 1:**
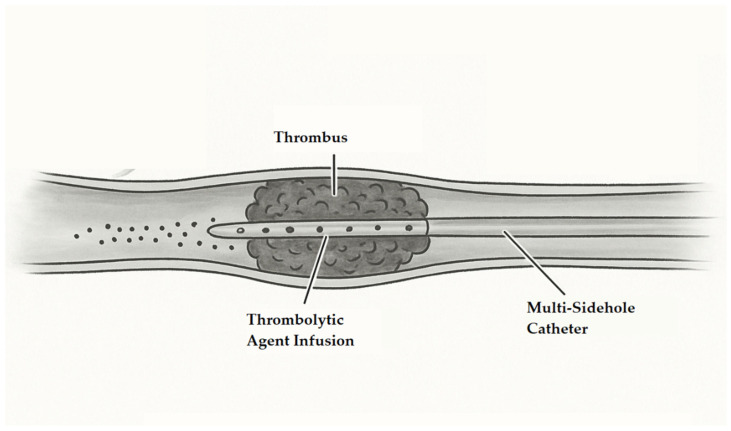
Schematic illustration of CDT. The multi-sidehole catheter is inserted into the thrombosed segment of the deep vein, allowing for localized delivery of the thrombolytic agent.

Patients in the CDT group underwent ultrasound-guided venous access and catheter placement. The popliteal vein was determined as the main access route due to factors such as increasing experience over time, the ease of ultrasound-guided vascular access, and the shortness of the procedure. In cases where the popliteal vein was not suitable, the femoral vein or the vena saphena parva were used as alternative access routes. Small saphenous vein catheterization was preferred in cases where the popliteal vein was deep or the attempt was unsuccessful. In popliteal vein catheterization, patients were placed in the prone position and a 5F catheter was inserted into the popliteal vein under ultrasound guidance. A 0.035 inch hydrophilic guidewire was then advanced along the thrombotic segment, and a multiple sidehole infusion catheter (Cook Medical, Bloomington, IN, USA) was inserted into the thrombotic lesion. In femoral vein catheterization, a 5F catheter was inserted into the femoral vein under ultrasound guidance in supine patients, and a multiple sidehole infusion catheter (Cook Medical, Bloomington, IN, USA) was positioned to reach the iliocaval junction for catheter-directed thrombolysis. Alteplase (ACTILYSE^®^ Boehringer Ingelheim, Germany) was infused through the catheter as a thrombolytic agent (Figure 1). The dose of alteplase was 20 mg of intravenous bolus followed by an intravenous infusion of 0.5–1 mg/h for 24 h. At the end of this period, the infusion was terminated regardless of the thrombus dissolution rate obtained. After the procedure, patients were closely monitored in the intensive care unit, and hemodynamic parameters and bleeding complications were evaluated. Moreover, complete blood count (CBC), the international normalized ratio (INR), aPTT, and serum creatinine levels were checked on a regular basis throughout the post-thrombolysis period. Anticoagulant therapy was initiated in all patients during the post-thrombolysis period and was continued for at least 3 months, with the duration individualized according to personal thromboembolic risk factors.

Bleeding complications were monitored for 96 h after the procedure. During the post-discharge follow-up period, patients were invited to outpatient clinic visits at regular intervals. The first follow-up visit was performed 1 month post-discharge, followed by follow-up visits at 3, 6, and 12 months. All 175 patients were evaluated at 30 days. Follow-up data at 3, 6, and 12 months were available for 159 (90.8%), 142 (81.1%), and 123 (70.3%) patients, respectively. During the follow-up period, clinical symptoms, DVT-related complications, and the development of post-thrombotic syndrome were evaluated. At each follow-up visit, in addition to physical examination, the venous system was evaluated by Doppler ultrasonography (USG) for the presence of recanalization and recurrent thrombosis. Patients’ compliance with anticoagulant therapy was also evaluated.

Anticoagulant therapy was ceased once thrombus removal reached at least 90% and was recorded as “thrombus removal rate”. The thrombus removal rate was defined as a ≥90% reduction in thrombus burden based on Doppler ultrasonography findings showing near-complete recanalization of the affected venous segment and restoration of venous flow. In our study, thrombus resolution was assessed at predefined intervals using Doppler ultrasonography. This imaging modality was preferred due to its non-invasive nature and its capability to provide a visual evaluation of venous blood flow and the presence of thrombus. During the follow-up period, thrombus resolution was defined based on the recanalization of thrombosed segments and the recurrence of thrombosis.

### 2.4. Statistical Analysis

Statistical analyses were conducted to evaluate differences between the two treatment groups—AC and CDT—across several outcome measures. Continuous variables were expressed as mean ± standard deviation (SD), providing a measure of central tendency and variability for the data. These variables were compared using the *t*-test, which is appropriate for comparing means between two independent groups. The *t*-test assumes normal distribution and equal variance between groups; in this study, the Shapiro–Wilk test was used to assess normality and Levene’s test to evaluate homogeneity of variances.

Categorical variables, which include outcomes such as the incidence of complications (e.g., gastrointestinal bleeding, PE), were presented as frequencies and percentages. For comparisons between these categorical variables, Fisher’s exact chi-squared test was employed. The chi-squared test is commonly used to assess associations between two categorical variables, but in cases where the expected cell frequencies are low (typically less than 5), Fisher’s exact test provides a more accurate result. This ensures the validity of statistical inference, especially when working with small sample sizes or sparse data in contingency tables.

The IBM SPSS version 20.0 software program was used to conduct the analyses, a well-established statistical tool in medical research. For all statistical tests, a *p*-value < 0.05 was considered indicative of statistical significance, meaning there was strong evidence to reject the null hypothesis that no difference exists between the groups.

## 3. Results

In total, 175 patients with DVT were included in the study, of which 98 were given AC and 77 received CDT. Baseline demographics of both the groups were similar to one another, enabling a fair comparison between the treatment strategies (Table 1).

The PE rate was comparable in the two groups, as it was observed in 8.1% of the AC group and 7.7% of the CDT group (*p* = 0.332). Of the total 14 PE events recorded in the study, 13 were identified at the time of initial DVT diagnosis. One PE occurred during follow-up at the 3-month visit in a patient from the AC group. The occurrence of gastrointestinal bleeding (2% vs. 3.8%, *p* = 0.789) and intracranial hemorrhage (1% vs. 3.8%, *p* = 0.45) did not differ significantly too, demonstrating that CDT did not increase the risk of major bleeding complications. Hematoma occurred in 1% of AC patients and 2.5% of CDT patients (*p* = 0.988), once more validating the safety profile of both therapies. Mortality was low in both groups (1% in AC vs. 2.5% in CDT, *p* = 0.707) and did not differ statistically significantly (Table 2). The mean duration of anticoagulation was 5.3 ± 1.1 months in the CDT group and 5.6 ± 1.3 months in the AC group, with no statistically significant difference (*p* = 0.47).

Thrombus distribution analysis revealed no statistically significant difference in both groups. The most frequently affected venous segments were the femoral vein (79.5% in AC vs. 80.5% in CDT, *p* = 0.189) and the popliteal vein (74.4% in AC vs. 66.2% in CDT, *p* = 0.307). Iliac vein thrombosis was more common in the CDT group (58.4% vs. 41.8% in ACA, *p* = 0.705), while calf vein involvement was present in approximately one-third of the patients in both groups (33.6% vs. 27.2%, *p* = 0.605). Inferior vena cava thrombosis was not very prevalent but was present in 4% of the AC patients and 6.4% of the CDT patients (*p* = 0.669) (Table 3).

One of the principal findings of the study was the significantly higher rate of thrombus removal in the CDT group, particularly in iliac vein thrombosis. It was also observed that the clinical symptoms resolved more quickly in this group. Among the regularly evaluated clinical presentations—edema of the extremities, pain, and sensation of weight—the median time to symptom relief was 4.3 ± 1.7 days in the CDT group compared to 7.8 ± 2.2 days in the AC group (*p* = 0.018). This difference was discovered to be of statistical significance, indicating a more rapid symptomatic improvement related to CDT. In the CDT group, impressive thrombus removal was achieved right after the 24 h alteplase infusion, as supported by post-procedure venous imaging. In the AC group, however, thrombus resolution developed more gradually and was most pronounced at the 3-month follow-up. Thrombus resolution was achieved in 97.7% of CDT patients compared with 78% in the AC group (*p* = 0.003), which was a clear advantage of CDT in this vascular territory. Although the rates of thrombus removal were also slightly higher in the CDT group for both femoral (91.1% vs. 92.3%, *p* = 0.081) and popliteal veins (80.3% vs. 91.7%, *p* = 0.318), there were no significant differences. Clearance of the thrombus of calf veins was similar in both groups (81% in AC vs. 85.7% in CDT, *p* = 0.345). Interestingly, in patients who had inferior vena cava thrombosis, complete thrombus removal was observed in all cases regardless of treatment modality (100% in both groups, *p* = 0.869) (Table 4). This would suggest that for more central thrombi, both CDT and ACA can be very effective.

During routine follow-up evaluations at 1, 3, 6, and 12 months, all patients underwent Doppler ultrasonography to assess thrombus status. Residual thrombus was identified in 11 patients (11.2%) in the AC group and in four patients (5.2%) in the CDT group. Additionally, recurrent thrombotic events were observed in three patients (3.1%) in the AC group and in one patient (1.3%) in the CDT group. Doppler ultrasonography was conducted at 1, 3, 6, and 12 months post-treatment to quantify the temporal resolution of thrombus. For the CDT group, complete resolution of thrombus occurred in 90.9% of cases at one month, 88.3% at three months, 87.0% at six months, and 85.7% at twelve months. Complete resolution in the AC group was found in 81.6%, 78.2%, 76.5%, and 73.5% of cases at the same time intervals, respectively.

## 4. Discussion

Conventional anticoagulant therapy, the efficacy of interventional procedures such as CDT, and mechanical thrombectomy for the management of DVT have been investigated for a long time. The results of our research, in conjunction with the existing literature, will be of great importance in determining the efficacy and complication rates of the different techniques.

CDT is also known to be a highly effective technique for prevention of the development of PTS, particularly in acute iliofemoral DVT patients with a low risk of major bleeding and in patients with acute iliofemoral DVT (<14 days) and low risk of bleeding. The CAVENT study proved that target vessel patients were maintained better and the rate of PTS decreased among patients treated with CDT [12]. Widespread application of CDT might be hindered by major bleeding caused by thrombolytic agents. The ATTRACT study clarified that although CDT alleviated symptoms of thrombosis in the acute phase, it did not have any major effect on the occurrence of PTS in the long run [13]. These findings emphasize that CDT should be employed with appropriate patient selection.

Consistent with our results, a major meta-analysis of 10 clinical trials conducted by Lu et al. concluded that CDT was associated with significant improvement in iliofemoral vein patency and a decreased risk of severe PTS when compared with anticoagulation therapy alone [14]. Benefits regarding the prevention of mild or overall PTS were found to be inconclusive because of significant heterogeneity between the included studies. The meta-analysis further implied a high incidence of bleeding and pulmonary embolism in the CDT group, as also identified in the safety profile in our population. In spite of these associated risks, nonetheless, the improved thrombus clearance and symptom improvement exhibited by our CDT patients indicate the potential for the use of CDT in specific patient populations, in accordance with this meta-analysis.

Mechanical thrombectomy is now a much sought after treatment for DVT. The DEFIANCE trial showed that mechanical thrombectomy provided faster clot removal compared to anticoagulation therapy alone but was not linked with a considerable difference in long-term venous patency and onset of PTS [15]. However, other research has pointed out that the addition of CDT to mechanical thrombectomy can produce better clinical outcomes [16,17].

As indicated in Makedonov et al.’s study, pharmacomechanical thrombolysis, especially when combined with endovenous methods, not only reduces clot clearance time but also minimizes bleeding risk through the reduction in the systemic thrombolytic dose [18]. The intervention has the additional advantage of the long-term preservation of venous function, especially in active and young patients. The research demonstrated reduced PTS rates compared to conventional anticoagulation therapy. These findings suggest that the addition of mechanical procedures to CDT can enhance its efficiency even further.

Ultrasound-guided interventional procedures also have an important part to play in the treatment of DVT. It has been shown that ultrasound-guided interventions can be better than conventional methods in terms of preserving venous patency [5,19]. Some studies have also shown that early intervention procedures are associated with lower rates of PTS [7,20]. As per the study by Thukral et al., the application of early endovenous treatments in DVT management was emphasized to realize not just symptom alleviation but also a significant effect on quality of life. The study is particularly relevant because of its potential to reduce hospitalization and total health costs. It was further mentioned that these interventions could be particularly effective in the iliofemoral segment with minimal complication rates. This would mean that invasive interventions are not merely economically but also clinically reasonable options [20].

The results of our research emphasize patient-specific tailored treatment approaches in accordance with the literature, especially in patients at high risk for thrombotic burden progression or recurrent DVT events—such as those with extensive proximal thrombosis or prior VTE—interventional therapies such as CDT and mechanical thrombectomy should be carefully selected, and the long-term results of these methods should be investigated [2,18]. In addition, patient compliance and the persistence of anticoagulant therapy on the follow-up of DVT are significantly important to reduce recurrence rates [3,20]. Dicks et al.’s study shows that the individualization of interventional treatment options by using sophisticated imaging techniques can optimize treatment outcomes while also reducing complication rates [19].

In the paper, it was stated that diagnostic tools like ultrasound and MR venography utilized in the management of DVT have a determining role in the selection of patients and provide significant contributions particularly in the early diagnosis of complications like subclinical pulmonary embolism. In this regard, it is realized that not just the forms of treatment but also the supportive methods applied in the decision-making process must be optimized.

The global burden on health caused by DVT should also be considered. DVT prevalence is seen to be on the increase, and VTE remains one of the leading causes of morbidity and mortality across the world [1,19]. This once again highlights the importance of early diagnosis and optimal treatment practices. Individualized approaches are of paramount importance in the management of DVT. A management strategy needs to be developed based on clinical status, risk factors, and patient profile. Interventional techniques such as anticoagulant therapy, CDT, and mechanical thrombectomy can each be beneficial in selected patient groups. However, long-term follow-up trials in larger patient groups are needed to further understand the efficacy and long-term outcomes of these methods.

### Study Limitations

Our study has some limitations. First of all, its retrospective design limits causal relationships. The single center of the study may also affect the generalizability of the results to other institutions or regions. Second, the lack of randomization may lead to some decisions that affect treatment decisions. As the follow-up period was up to 12 months, it is difficult to detect long-term complications such as post-thrombotic syndrome. Another reason is that our study excluded high-risk patients, which limits its applicability to these populations. Another limitation is the lack of subgroup analysis by DVT etiology (provoked vs. unprovoked), which can influence recurrence risk along with therapeutic choices. Future studies with larger sample sizes can rectify this valuable clinical variable. Lastly, inconsistencies may have occurred due to variability in CDT procedural techniques, affecting the comparability of the results.

## 5. Conclusions

The optimal approach to DVT treatment should be determined according to the individual risk factors and clinical condition of the patient. Although interventional methods may be effective in certain patient groups, they may not be appropriate for all patients. Therefore, future large-scale and long-term studies will contribute to a better understanding of the efficacy of treatment methods. Our study is important in terms of contributing to the existing literature and reveals issues that need to be supported with larger patient populations in the future.

## Figures and Tables

**Table 1:** Baseline characteristics of the patients.

Characteristics	AC (*n* = 98)	CDT (*n* = 77)	*p*-Value
**Age (Year, Mean)**	57.2	54.3	0.954
**Male**	44 (44.8%)	35 (45.4%)	0.501
**Diabetes Mellitus**	10 (10.2%)	7 (9%)	0.687
**BMI > 30**	19 (19.3%)	22 (28.5%)	0.32
**Hypertension**	41 (41.8%)	32 (41.5%)	0.345
**Cerebrovascular Accident**	3 (3%)	2 (2.5%)	0.144
**Smoking**	33 (33.6%)	24 (31.1%)	0.309
**Hyperlipidemia**	23 (23.4%)	22 (28.5%)	0.211
**Malignancy**	9 (9.1%)	8 (10.3%)	0.103
**Previous DVT**	9 (9.1%)	5 (6.4%)	0.077
**Thrombophilia**	20 (20.4%)	13 (16.8%)	0.193

**Table 2:** Outcomes of patients undergoing AC or CDT groups.

Outcome	AC (*n* = 98)	CDT (*n* = 77)	*p*-Value
**Pulmonary Embolism**	8 (8.1%)	6 (7.7%)	0.332
**Gastrointestinal Bleed**	2 (2%)	3 (3.8%)	0.789
**Intracranial Hemorrhage**	1 (1%)	3 (3.8%)	0.45
**Hematoma**	1 (1%)	2 (2.5%)	0.988
**Death**	1 (1%)	2 (2.5%)	0.707

Data are presented as *n* (%) or mean ± standard deviation. AC: anticoagulation therapy alone; CDT: catheter-directed thrombolysis.

**Table 3:** Thrombus localization.

Lesion	AC (*n* = 98)	CDT (*n* = 77)	*p*-Value
**Inferior Vena Cava**	4 (4%)	5 (6.4%)	0.669
**Iliac Vein**	41 (41.8%)	45 (58.4%)	0.705
**Femoral Vein**	78 (79.5%)	62 (80.5%)	0.189
**Popliteal Vein**	73 (74.4%)	51 (66.2%)	0.307
**Calf Vein**	33 (33.6%)	21 (27.2%)	0.605

Data are presented as *n* (%) or mean ± standard deviation. AC: anticoagulation therapy alone; CDT: catheter-directed thrombolysis.

**Table 4:** Thrombus clearance rate by segment.

Lesion	AC (*n* = 98)	CDT (*n* = 77)	*p*-Value
**Inferior Vena Cava**	4 (100%)	5 (100%)	0.869
**Iliac Vein**	32 (78%)	44 (97.7%)	0.003
**Femoral Vein**	72 (92.3%)	57 (91.1%)	0.081
**Popliteal Vein**	67 (91.7%)	41 (80.3%)	0.318
**Calf Vein**	27 (81%)	18 (85.7%)	0.345

Data are presented as *n* (%), representing the proportion of patients with successful thrombus clearance as assessed by venous Doppler ultrasonography. For the CDT group, clearance was assessed within 24 h after completion of thrombolysis. For the AC group, clearance was assessed at the 3-month follow-up visit. AC: anticoagulation therapy alone; CDT: catheter-directed thrombolysis.

## Data Availability

Data are contained within the article.
